# Radiation‐induced tissue damage and response

**DOI:** 10.1002/path.5389

**Published:** 2020-02-21

**Authors:** William H McBride, Dörthe Schaue

**Affiliations:** ^1^ Departent of Radiation Oncology University of California, Los Angeles (UCLA) Los Angeles CA USA

**Keywords:** clonogen, ionizing radiation, stem cell, oxidative stress, acute and late responsessenescenceautophagyinflammationregeneration

## Abstract

Normal tissue responses to ionizing radiation have been a major subject for study since the discovery of X‐rays at the end of the 19th century. Shortly thereafter, time–dose relationships were established for some normal tissue endpoints that led to investigations into how the size of dose per fraction and the quality of radiation affected outcome. The assessment of the radiosensitivity of bone marrow stem cells using colony‐forming assays by Till and McCulloch prompted the establishment of *in situ* clonogenic assays for other tissues that added to the radiobiology toolbox. These clonogenic and functional endpoints enabled mathematical modeling to be performed that elucidated how tissue structure, and in particular turnover time, impacted clinically relevant fractionated radiation schedules. More recently, lineage tracing technology, advanced imaging and single cell sequencing have shed further light on the behavior of cells within stem, and other, cellular compartments, both in homeostasis and after radiation damage. The discovery of heterogeneity within the stem cell compartment and plasticity in response to injury have added new dimensions to the consideration of radiation‐induced tissue damage. Clinically, radiobiology of the 20th century garnered wisdom relevant to photon treatments delivered to a fairly wide field at around 2 Gy per fraction, 5 days per week, for 5–7 weeks. Recently, the scope of radiobiology has been extended by advances in technology, imaging and computing, as well as by the use of charged particles. These allow radiation to be delivered more precisely to tumors while minimizing the amount of normal tissue receiving high doses. One result has been an increase in the use of schedules with higher doses per fraction given in a shorter time frame (hypofractionation). We are unable to cover these new technologies in detail in this review, just as we must omit low‐dose stochastic effects, and many aspects of dose, dose rate and radiation quality. We argue that structural diversity and plasticity within tissue compartments provides a general context for discussion of most radiation responses, while acknowledging many omissions. © 2020 The Authors. *The Journal of Pathology* published by John Wiley & Sons Ltd on behalf of Pathological Society of Great Britain and Ireland.

## Introduction

Within weeks of Röntgen's discovery of X‐rays in 1895 1, many workers developed dermatitis from using low power X‐ray tubes to try to reproduce his findings. A few years later, Becquerel showed that natural sources of radioactivity can also cause inflammatory skin burns. Unlike thermal burns, radiation burns develop after a characteristic latent period, an observation that prompted Pierre and Marie Curie to self‐experiment on the dose relationships for latency and persistence of radium‐induced lesions. The latency of inflammation in the human skin was in fact sufficiently predictable to calibrate radiation tubes for clinical dosimetry using the minimal erythematous dose as the unit. In 1905, Heineke 2 noted that the chronology of latencies for different tissues was relatively constant across species, even though Miescher 3 noted several ‘waves’ of erythema in human skin that Pohle 4 later attributed to radiation‐induced changes in capillary density. Early histopathological observations on the vascular effects of ionizing radiation (IR) documented swelling and degeneration of endothelium and capillary occlusion 5, hyperemia and exudation of serum and red cells 6, capillary leakage 7 and inhibition of vascular capillary budding 8. A lengthy debate began as to the importance of vascular radiation damage for loss of tissue function that continues to this day 9. Although vascular responses are clearly relevant, in general the radiation response of different adult tissues is best explained by their diversity of structure and endogenous stem/progenitor cell content, which are the major thrust of this review.

Heineke 10 was the first to point to tissue differences in time–dose responses, contrasting the very rapid appearance of radiation‐induced lymphopenia with the 2‐week latency of severe dermatitis, and with the relative lack of changes in liver and kidney over the same time period. He also noted that lymphocytes died within a few hours and were cleared by phagocytes that appeared in tissues in large numbers 11. Differences in radiosensitivity between cell populations within a tissue were highlighted by Regaud and Blanc's 12 detailed histological descriptions of spermatogenesis. Bergonie and Tribondeau 13 condensed findings at that time into a ‘law’, saying essentially that ‘the effects of irradiation on cells are more intense the greater their reproductive activity’ 14. This holds some truth, but there are many exceptions, not the least being that some non‐dividing cells, such as small lymphocytes, are very radiosensitive, dying by apoptosis. Regaud and Nogier 15 went on to show that three radiation doses given 15 days apart could sterilize rams without damaging the scrotum, indicating that differences in proliferative potential between tissues could be exploited by dose fractionation. This has remained a central thesis of cancer radiation therapy (RT) until recent times, with 1.8–2 Gy per fraction given daily, 5 days a week for 5–7 weeks becoming ‘conventional’ treatment.

The optimization of RT for cancer clearly needed to be understood to determine how best to manipulate the time, dose and size of dose per fraction to exploit differences between individual normal tissues and tumors. This was aided by the advent of *in vitro* clonogenic assays 16 and *in vivo* colony‐forming assays developed by Till and McCulloch 17. Considered to be the ‘fathers of stem cell science’, Till and McCulloch showed that bone marrow cell transfer in mice could prevent lethality after whole body irradiation (WBI) and that the radiosensitivity of the stem cells could be assessed *in vivo* by their ability to form colony‐forming units in the spleen (CFU‐S) after transfer. Withers extended the stem cell approach by developing *in situ* clonogenic assays for radiation responses in skin 18, jejunum 19, colon 20, testes 21 and kidney 22, whereas Jirtle *et al* 23 used an *in vivo* transfer system to quantify radiation responses of hepatocytes. Functional assays were developed for other tissues, including lung (pneumonitis and fibrosis), spinal cord (paralysis), wound healing (breaking strength), mucosa (inflammation) and hair follicles (epilation). Withers and coworkers 24 developed an isoeffect formula that became the most popular way to compare these clonogenic and functional endpoints in different tissues based on simple linear (α) and quadratic (β) components. By plotting isoeffect curves for fractions of different sizes, they showed that endpoints for late responding normal tissues changed more dramatically with size of dose per fraction than those for acute tissues (up to 6 weeks after RT); a difference that could be described by α/β ratios 25. Withers 26 also summarized the biology behind dose fractionation effects by the 4Rs. (1) Repair of sublethal damage that spares late responding tissues with slow turnover, e.g. CNS. (2) Redistribution into the radiosensitive G2/M phase of the cell cycle that spares tissues with slow turnover. (3) Repopulation/regeneration that spares normal tissues with rapid turnover that can proliferate during a fractionated course, e.g. mucosa. (4) Reoxygenation that decreases the hypoxic radioresistant fraction within tumors. These principles have guided clinical radiation oncologists for decades and prompted successful clinical trials for fractionation schemes that deviated from the classical 2 Gy per fraction 27.

This review will focus on how structural diversity between adult tissues impacts their radiation responses and how plasticity within tissue compartments might impact their regeneration. We are not able to consider, in full, important issues like dose, dose rate and the quality of radiation, or non‐adult tissues. Low‐dose stochastic effects will be sacrificed for cover of deterministic effects. Much of the relevant radiobiology is derived from animal models using photon irradiation delivered to fairly large fields, but we will briefly comment on how new technologies entering the clinic for cancer RT might change existing paradigms.

## Tissue diversity and response

Tissues vary greatly in both their tolerance to radiation and in their latency, which is determined largely by the tissue turnover. For example, in C3H mice, 14 Gy X‐rays deplete epithelium in about 3 days in the jejunum, 5 days in the colon, 10 days in the stomach, 12–24 days in the skin, 30 days in seminiferous tubules of the testis and 300 days in kidney tubules 22. Stem cells have also been reported to turnover at very different rates in different tissues under steady‐state conditions 28. Under homeostatic conditions, by definition, the rate of cell production equals the rate of cell loss, i.e. the cell loss factor (ϕ) = 1.0. Asymmetric division to give cell loss may be a property of individual ‘stem’ cells or of a population that stochastically produces on average 50% ‘stem’ and 50% differentiating cells 28, 29; properties that may be autonomous or mediated through a supportive ‘niche’, a concept first defined in 1978 30. Regeneration occurs after cell loss and requires an increase in cell production and a decrease in ϕ to <1.0.

**Figure 1:**
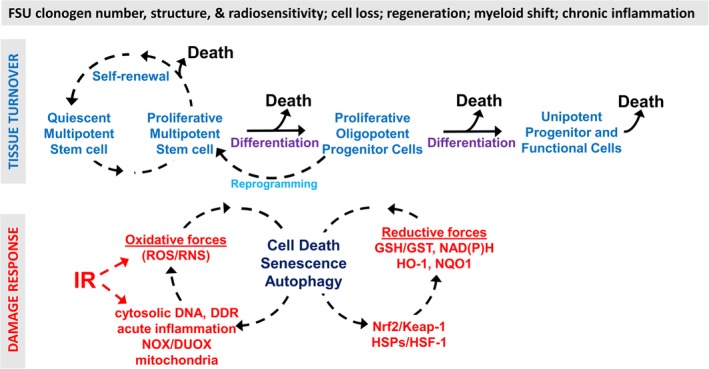
Some of the features defining most normal tissue radiation responses. A general outline of the turnover of cell populations in normal tissues is presented in the upper panel. In the lower panel, IR triggers damage responses that set in motion recurring cascades of oxidative/reductive forces that aim to restore function but instead cause further cell death, senescence and autophagy, and immune involvement in the form of a myeloid shift and chronic inflammation.

The advent of lineage tracing technology, advanced imaging and single cell sequencing has improved the accuracy with which the behavior of stem, and other, cellular compartments in tissues can be studied. These are reviewed elegantly elsewhere 28. The consensus is that there is a ‘continuum of stemness’ with considerable steady‐state heterogeneity in numbers and organization, as well as ‘plasticity’, which includes reprogramming of more differentiated cells towards stemness and that could be particularly relevant in radiation‐induced regeneration 31, 32. Given this heterogeneity, we will use the term ‘stem’ cell loosely, rather than attempt to define it with respect to specific markers, which may be misleading 28. The basic cellular elements present in most normal tissues, and the effects of IR, are summarized in Figure 1. In a sense, these recent findings of stem cell heterogeneity strengthen the relevance of *in situ* clonogenic assays as measures of radiation response, as the term ‘clonogen’ can be taken to represent a functional regenerative unit that implies stem cell involvement but without prejudice.

In addition to the variables noted above, differences between tissues with respect to radiosensitivity and volume effects may also be explained by their organization into functional subunits (FSU), where a FSU is the volume that can be regenerated from one surviving clonogen 33. For example, epilation may occur after a lower radiation dose than desquamation simply because hair follicles have a smaller number of clonogens/FSU 34. A FSU may correspond to a structural element, for example a tubule, or may not, for example, it may depend on how far a clone can migrate. If FSUs are arranged in series, like links in a chain, as in the spinal cord or nerves, tissues will probably demonstrate a strong, high dose–volume effect over a short distance. If arranged in parallel, as in skin and liver, the tissue will be better able to tolerate high doses to small volumes but may be susceptible to low doses to large volumes as function will be determined largely by the volume not irradiated. Tissue tolerance to IR is therefore a function of both the number and the radiosensitivity of clonogens in a FSU, and the number and organization of FSUs in a tissue. This analysis requires that normal tissues have stem cells or clonogenic regenerating units, which will now be discussed with respect to tissue turnover.

### Acute responding tissues

Acute responding tissues, like intestine, bone marrow, skin and testes, turn over rapidly and have well‐defined stem cell populations, at least a portion of which actively cycle under steady‐state conditions. However, responses of these tissues to IR vary considerably, as does their ability to regenerate. Regeneration can be measured by split‐dose experiments, i.e. the size of a second dose needed to negate the effect of prolongation in overall treatment time. In such experiments, the jejunum recovers rapidly and well, with the replicating pool being regenerated prior to differentiation being resumed; ϕ becomes close to zero. For skin, ϕ becomes closer to 0.5, whereas regeneration in the testes is very slow and ϕ is little altered. Sperm counts remain low for a long time after exposure. These differences are relevant to the fractionation schedule used clinically and to the retreatment of lesions with RT, but also mean that each tissue must be considered on its merits.

The jejunum turns over very rapidly. The crypt houses a niche where basal columnar epithelial stem cells (BSC) reside. At least some BSCs cycle daily and give rise to rapidly proliferating progenitor/transit amplifying cells that differentiate into mature intestinal epithelial cells that journey up the villus and are shed into the lumen 35. BSCs that express the R‐spondin receptor and Wnt target molecule LGR5 are interspersed between Paneth cells, which, together with crypt structure, mesenchymal fibroblasts, endothelium, pericytes and other cell types, nurture and protect the stem cell population by providing Notch, WNT and BMP family members, growth factors such as EGF and cytokines such as IL‐11 and IL‐22 36.

After 19 Gy total abdominal irradiation, which is lethal for 50% of defined‐flora C3H mice by 5–10 days, jejunal clonogenic cell survival is decreased to about 8 × 10^–6^ 37. The colon is generally more radioresistant and responds later than the small intestine 20, 38. Radiosensitivity varies with the strain of mouse, the length along the intestine, the proximity of Peyer's patches 39 and the microbial flora, as irradiation can promote bacterial translocation across the intestinal barrier 40. Furthermore, intestinal acute radiation syndrome (iARS) after WBI requires a lower dose than after total abdominal irradiation, a difference that can be neutralized by transfer of immunohematopoietic cells, suggesting ‘accelerated’ hematopoietic ARS as a potential cause of death 37, 41. The LGR5^+^ BSCs have been reported variably as radiosensitive 42 and radioresistant 43, but they are generally depleted from the crypt by 3.5 days after 14 Gy and clonogenic epithelial foci begin to appear from a radioresistant subset. In 1977, Potten 44 suggested that these reside at position +4 just above the uppermost Paneth cell; a population we now know to be LGR5^−^ and Bmi1^+^ 42. Mindful of the pitfalls involved in the use of markers 45, the data suggest that a Bmi1^+^ radioresistant reserve stem cell pool, or at least a reprogrammable plastic population, restores LGR5^+^ cells in the crypt niche as well as the radiosensitive transit amplifying compartment following irradiation exposure 42, 46. It should be noted that reprogramming may be limited by p53‐mediated DNA damage responses (DDR) 47 and it is dose and time dependent, senescence sensitive 32 and influenced by non‐targeted effects 48.

Bone marrow, under steady‐state conditions, contains primitive hematopoietic stem cells (HSC) that are functionally and molecularly heterogeneous but within which is a rare population with unique capability for self‐renewal and multilineage differentiation 49. HSCs are held in niches under the influence of various adjacent stromal cells that present bound or secreted molecules, including the classic hematopoietic CSFs, CXCL12, pleiotropin and many other signals 50, 51. Several niches have been proposed, but differential expression on lineage‐negative HSC of signaling lymphocytic activation molecule (SLAM) family receptors, such as CD150, CD48 and CD244, has most clearly defined a vascular niche with HSCs in proximity to arterioles and sinusoids 52. Numerous influences on HSC behavior by factors derived from endothelium, pericytes and fibroblasts have been described 51. Indeed, there is growing evidence for a hemangioblast stem cell that can give rise to both HSCs and endothelial progenitor cells 53. At least those HSC capable of long‐term lineage reconstitution in radiation‐conditioned hosts (LT‐HSC) are largely non‐cycling and stay in the bone marrow 54, but produce low levels of progenitor cells that are released into the circulation under normal conditions. Egress is achieved through a complex interplay of cytokines, chemokines and adhesion molecules, particularly involving the SDF‐1/CXCR4 axis, purinergic signaling and phosphosphingolipids 50. As HSCs differentiate they follow a hierarchical ‘age’ structure with progressive restriction in lineage and self‐renewal (Figure 1), which is reflected in the changing composition of exogenous CFU‐S on days 5, 8 and 11 after marrow transfer into irradiated hosts 55. There is also evidence of a self‐sustaining myeloerythroid progenitor population 56 that acts as an emergency reserve of immature and maturing leukocytes for rapid mobilization in response to challenge, such as after WBI.

Radiation responses in the hematopoietic system are inevitably complex. Circulating lymphocytes are very radiosensitive, dying largely by apoptosis. Levels drop rapidly, even after local irradiation as cells circulate through the radiation field. Circulating myeloid cells by contrast can transiently increase in number within hours after local IR or even lethal WBI, forming part of an emergency mobilization response 57. Precursors appear in the circulation that express both neutrophil and macrophage markers 58. These migrate into many tissues, irradiated or not, under the influence of CSF1 59 and perhaps CCL2, IL‐6 and IFN‐α/β pathways 58, 60. Later overshoots in the production of myeloid cells in the circulation have been described in many species 57. Quiescent LT‐HSC are relatively radioresistant, but appear to be very sensitive to oxidative stress, which can be generated by even low (0.02 Gy) doses of IR 61, generated directly or through proinflammatory pathways (Figure 1). Radiation‐induced senescence is a likely outcome of radiation‐induced oxidative stress, as is autophagy 62, 63. Proinflammatory responses are controlled by reductive pathways, especially those under Keap1/Nrf2 control, that critically regulate LT‐HSC levels 61, 64. Nrf2 also regulates PU.1, the master myeloid cell regulator 65, probably through NAD(P)H:quinone oxidoreductase, whose loss leads to myeloid hyperplasia 66. However, a common result is a long‐term defect in HSC reconstituting ability and continuing cycles of oxidative and reductive stress 61, 67 and inflammation, with a myeloid shift; a pattern that is repeatedly reinforced 68, 69. These chronic inflammatory events are a hallmark of late radiation effects, including life shortening 69. G‐CSF 70 and probably other inflammatory stimuli can exacerbate the HSC defect, which raises questions as to the use of CSFs as mitigators of radiation damage. These radiation‐induced changes appear to be remarkably similar to aging with its LT‐HSC defects 71, and upregulation of genes specifying the myeloid cells at the expense of those of the lymphoid system 72. Perhaps a myeloid shift is the price we pay for having a self‐sustaining, partly autonomous myeloerythroid progenitor population that responds rapidly to injury to initiate tissue repair 56. This population is sufficient to protect against hematopoietic acute radiation syndrome (hARS) in mice and is responsible for the day 8 CFU‐S population 73. Rapid recovery of this progenitor population may allow sufficient time for the regeneration of other hematopoietic compartments with slower turnover. Certainly, promoting this population results in mitigation of hARS and other WBI syndromes 58. Not surprisingly, this is dose‐dependent and after higher WBI doses accelerated hARS can occur, which is presumably due to critical loss of a more differentiated progenitor cell population.

In the skin and mucosa, squamous epithelia proliferate solely in the basal layer and cell loss is determined largely at the population level 28, 74. Progeny committed to differentiate migrate upwards to form a layer of keratinocytes that are then shed. After IR exposure, cells lost from the proliferating compartment are regenerated by a shift towards symmetrical division and differentiation decreases, although shedding continues unabated. The extent of clonogenic cell depletion determines the outcome. Survival of >10^−6^ clonogens per cm^2^ is needed to prevent radiation‐induced moist desquamation in mouse skin 18. Proliferating clones can often be seen in skin and mucosa of patients during conventional RT. The mucosa begins to repopulate 10–12 days after the initiation of treatment and this increases the tolerance of the tissue by at least 1 Gy/day, equivalent to a doubling of clonogens every 2 days 18. Other epidermal stem cell pools exist in the skin in follicles, glands and other sites that are competent to differentiate along all epidermal lineages. Although, normally, their involvement is restricted to their own lineage their additional plasticity may manifest in injury situations 28.

In testes, Regaud 12 showed that radiosensitivity decreased with differentiation, radiating from spermatogonia on the periphery to spermatids in the center of the seminal tubules 16. Spermatogenesis is very radiosensitive, with transient infertility around doses above 0.1 Gy that is permanent at 5–8 Gy 75. Spermatogenic stem cells cycle continuously, but slowly, in the basal layer of the tubules, appearing to divide symmetrically before differentiating into mature haploid spermatozoa, a process that takes about 70 days. Single cell tracing in mice of GFRalpha1+ stem cells *in vivo* suggests an additional dynamic dimension where syncytial spermatogonia can contribute to stem cell function in homeostasis. After irradiation, spermatogenesis is only partially restored and regeneration is poor 21, 76. Recovery is very slow, taking 1–2 years after 2–3 Gy, with a risk of azoospermia after higher doses 77.

### Acute radiation syndromes (ARS)

Dose–time lethality curves of iARS and hARS are fairly predictable. iARS occurs before hARS but requires a higher dose. Mortality occurs within a narrow dose window. Increasing the dose decreases latency slightly before a plateau is reached 41, 57. Mortality is generally due to loss of proliferative stem/progenitor cells that fail to provide functional cells, but other causes have been identified. For example, immunosuppression can allow bacterial translocation across the gut and sepsis with lethality that is generally earlier than normal and occurs after lower doses. Immunosuppression may resolve in a couple of months, but full immune reconstitution may take many months or years, or may never occur. Extensive skin damage can also contribute to morbidity and mortality. For example, about 20% of Chernobyl patients who developed ARS had skin lesions involving over 50% of their body surface; some had respiratory tract lesions, probably from isotope inhalation. A small number had beta burns as the primary cause of death 78.

In addition to iARS and hARS, a cerebrovascular/CNS syndrome (CVS/CNS‐ARS) can occur within a day or two after exposure to very high radiation doses (e.g. >20 Gy). This is associated with edema, hemorrhage and neutrophil infiltrates and, although some oligodendrocytes die by rapid radiation‐induced apoptosis 79, vascular damage is the most likely culprit, which may be through direct cell kill or radiation‐induced TNF‐α or VEGF 80.

A detailed description of radiation‐induced inflammation 81 is beyond the scope of this review, but is relevant to radiation‐induced tissue damage (Figure 1). In brief, although the initial wave of ROS generated by IR through radiolysis of water is over within 10^−3^ s, ROS levels remain high, being generated by biological processes; the main sources being damaged mitochondria and activated NADPH oxidases (NOX/DUOX). Classic ATM‐p53‐bax DDR cause ROS release from mitochondria. Immune DDR involves damage‐associated molecular pattern (DAMP) molecules released from damaged cells, like HMGB1 60, and cytoplasmic RNA and DNA 82. Toll‐like receptors, RIG‐1 (RNA) and cGAS/cGMP/STING (DNA) sensors connect DDR to proinflammatory responses, largely through NF‐κB and TKB1/IRF3 pathways, to activate positive and negative feedback loops for senescence, autophagy and cell death that perpetuate redox imbalances 60, 83, 84. Oxidative and reductive forces drive polar opposite phenotypes in the immune system and dictate the nature of the cytokines expressed and their role in normal tissue endpoints 85, including fibrosis 86. One consequence is the establishment of self‐sustaining periodic redox alterations and persisting cycles of tissue damage and inflammation. Radiation‐induced micronuclei are major sources of cytoplasmic DNA and activate the cGAS/cGMP/STING pathway. As these are produced during mitosis, they link the production of proinflammatory cytokines, especially type 1 IFN, to cell turnover. Furthermore, late effects in general are characterized by the presence of increasing chronic inflammatory responses that cause considerable morbidity, frailty and life shortening.

### Late responding tissues

The fairly distinct, if plastic, stem, progenitor, functional cell compartments with rapid turnover and acute responses to IR seen in hierarchical tissues 87 can be contrasted with late responding tissues with slow turnover, where it has been less easy to identify the contribution of stem cells to homeostasis and radiation‐induced regeneration. This distinction is important, as dose fractionation in the clinic spares slowly responding tissues more than those that show an early response.

With the exception of CNS‐ARS, the CNS is the classic late responding tissue. In adult brain, neural stem/progenitor cells have been identified in niches in the subventricular zone and in the dentate gyrus of the hippocampus. These are active sites of neurogenesis. Lineage tracing has shown that most stem/progenitor cells cycle and turnover slowly but can be activated to proliferate before migrating away to produce neurons or glia 88.

After adult brain irradiation with the equivalent of single doses of ~15–25 Gy, symptoms can appear in one or more phases; in days to weeks (acute phase), 1–6 months (subacute phase) or around 6 months or more (late phase). Acute and subacute symptoms are normally reversible, but late damage is progressive and more serious. Late effects have a predilection for white matter 89 but the histopathologic picture varies, including coagulation necrosis, vascular fibrinoid necrosis, edema and severe demyelination. Neurogenesis and proliferation in the hippocampus are inhibited by even low doses of IR 90. Progenitor cells seem more sensitive than quiescent stem cells and long‐term repopulation and recovery are slow 91, 92, 93. Even though transit amplifying cells have been reported to regenerate low‐dose irradiated (4 Gy) niches, suggesting some reprogramming can occur 88, the role of stem cells in recovery of the CNS is still controversial. The possible effects of irradiating the stem cell niches in patients with glioma have generated considerable discussion, with the possibility of generating neurocognitive defects being placed against the chances of the niche being the source of glioma stem cells, suggesting that increased radiation dose to the subventricular zone may be associated with longer progression‐free survival. From the beginning, opinions have been divided between glial and vascular origins of brain late effects. In general, higher doses tend to precipitate hemorrhagic necrosis that appears slightly earlier and at higher doses than severe neuronal loss following demyelination 94. Changes in cognition, including spatial and object recognition memory, fear conditioning and pattern separation behaviors have been ascribed to radiation‐induced defects in neurogenesis 91, 95 but, although important, these can be detected earlier and after lower doses than late demyelination, suggesting that they can be caused by neuroinflammation and oxidative stress. Proinflammatory cytokine expression occurs in mouse brain within minutes of cranial irradiation and thereafter pursues a rollercoaster path with further increases during the subacute and late periods that are associated with diffuse and severe demyelination, respectively, attempts at remyelination, and gliosis with immune cell infiltration and microglial activation 96, 97. TNFR2 is required for proliferation of neural progenitor cells 98 and its loss increases seizures in the brain 99, including after irradiation where subacute lethality is precipitated 80. Therefore, although there is evidence that radiation‐induced damage to the stem cell hippocampal niche can result in cognitive damage, its contribution to high‐dose late effects remain indirect.

The effects of irradiation on the kidney are most often measured functionally using filtration assays and indicate little recovery; previous irradiation can seriously compromise retreatment 100. Indeed, it remains controversial whether stem/progenitor cells actually exist in the mammalian adult kidney 101, but Withers and colleagues 22 showed that extensive tubular damage was the dominant lesion after irradiation, preceding glomerular sclerosis. Removing irradiated kidneys at 60‐68 weeks after exposure he found regenerating epithelialized tubules 60‐68 weeks after irradiation, the number declining logarithmically with dose.

Like the kidney, the liver has low turnover but can be stimulated to regenerate rapidly after surgery, leading to the general assumption that all mature hepatocytes are able to maintain homeostasis. However, lineage tracing in mice using Wnt‐responsive Axin2 identified a population of proliferating and self‐renewing diploid cells adjacent to the central vein in the liver lobule that could give rise to mature polyploid hepatocytes 102 and LGR5^+^ adult liver stem cells can be grown as organoids 103. The origin of the liver clones that grow on transplantation to recipient mice and whose radiation characteristics have been examined and found to be characteristic of a late responding tissue, is not known 23, 104.

## Future clinical relevance

An important premise in RT over the last century is that normal tissues (and tumors) vary in their response to dose fractionation, with late responding tissues with slow turnover being spared by fractionation more than early responding tissues. Although this is generally true for highly fractionated schedules, in recent years a variety of developments have been introduced into the radiation oncology clinic that aim to limit the amount of normal tissue within the radiation field. Although this is certainly desirable, the extent to which total tumor dose can be increased is debatable, as supralethal doses of radiation do not necessarily improve outcome 105. The impact of this technology on the responses of acute and late responding tissues and the 4Rs of dose fractionation is worthy of discussion.

CT/MRI imaging and faster computers have allowed intensity‐modulated RT with multileaf collimators shaping the beam to conform more closely to tumor shape while minimizing the amount of normal tissue exposed to high‐dose RT. Dose–volume histograms are produced that estimate the dose to different tissues. As a result, the use of hypofractionated regimens with RT given in one to five fractions has gained in popularity as it is more convenient for both patients and clinicians. Isoeffective doses for the change in size of dose per fraction can be estimated using linear (α) quadratic (β) exponents, although there is considerable uncertainty when extrapolating to high single doses. Hypofractionation makes sense, particularly for tumors that have a slow turnover in sites where there is little advantage from prolonged fractionation, e.g. prostate, and there is little to be gained from the enhanced recovery that the use of low doses per fraction bring late responding normal tissues. However, as stated earlier, it should be remembered that proliferation of acute responding normal tissues increases their radiation tolerance. Indeed, many regenerate faster than most tumors. As a result, shorter, more intense treatments can increase acute effects by not allowing enough time for their regeneration, even if the dose is calculated to be isoeffective for late effects and tumor. Also, volume effects, location and the organization of normal tissue FSUs become more critical when intensity‐modulated RT is used and there are many unanswered questions in this regard. Finally, the impact of hypofractionation on dose‐related inflammation and immune activation *in vivo* is still uncertain, even though it is known that some patients can generate tumor immunity that can be boosted by RT, something that has resulted in a large number of clinical trials combining RT with immunotherapy.

In a similar vein, charged particles, especially protons and carbon ions, have been introduced in some centers. Charged particles have a lower entry dose and form Bragg peaks where most of the energy is deposited. It is possible to spread out the Bragg peak to completely cover tumor with rapid fall‐off. The paths of charged particles are very different from photons and their relative biological effectiveness (RBE) is higher – dose for dose. The RBE for protons is only slightly more than for photons but carbon ions have far higher values. Again, dose corrections can be made for RBE, although this varies with the tissue and along the path, for example for protons it increases at the distal end of the Bragg peak. These uncertainties in the magnitude and location of high RBE radiations is a concern for late responding more than acute responding tissues.

FLASH RT is being tested where IR is delivered at an ultrahigh dose rate (>100 Gy/s) compared with conventional RT (∼0.1 Gy/s). FLASH RT appears to give neurocognitive benefits by decreasing oxidative stress and neuroinflammation 106. A full explanation has still to be established, but it may relate to alterations in the chemical interactions between ions and radicals in space and time, with different species being generated.

## Conclusions

Different tissues have different responses to IR. Tissues with rapid turnover and continuously cycling stem/progenitor populations respond acutely after exposure and regenerate rapidly. Such tissues show less effect of dose fractionation as long as regeneration is not compromised. Tissues with slow turnover respond late to IR and may have less dependence on stem/progenitor cells for regeneration and may rely more on proliferation and reprogramming of more mature cells. Chronic inflammation appears to play a greater role indirectly or directly in causing tissue failure. These differences are important in considering clinical RT and cancer treatment and may need further consideration with the rapid expansion into the clinic of novel technologies whose radiobiological effects are less well known. They are also relevant to mitigation of the effects of radiological exposure in accidents or terrorist action.

## Author contributions statement

The authors contributed equally to the writing of this paper.

## List of abbreviations

ARS, acute radiation syndrome; BSC, basal columnar epithelial stem cells; CFU‐S, colony‐forming units in the spleen; CNS‐ARS, central nervous system acute radiation syndrome; DAMP, damage‐associated molecular pattern; DDR, DNA damage response; FSU, functional subunit; hARS, hematopoietic acute radiation syndrome; HSC, hematopoietic stem cell; iARS, intestinal acute radiation syndrome; IR, ionizing radiation; LT‐HSC, long‐term hematopoietic stem cell; RBE, relative biological effectiveness; RT, radiation therapy; SLAM, signaling lymphocytic activation molecule; WBI, whole body irradiation.
